# Electron flow from PSII to PSI under high light is controlled by PGR5 but not by PSBS

**DOI:** 10.3389/fpls.2015.00521

**Published:** 2015-07-08

**Authors:** Mikko Tikkanen, Sanna Rantala, Eva-Mari Aro

**Affiliations:** Molecular Plant Biology, Department of Biochemistry, University of Turku, TurkuFinland

**Keywords:** regulation of photosynthetic electron transfer chain, cyclic electron transfer, thermal dissipation, NPQ, *trans*-thylakoid proton gradient, control of Cyt *b_6_f*, P700 oxidation

## Abstract

Absence of the Proton Gradient Regulation 5 (PGR5) protein from plant chloroplasts prevents the induction of strong *trans*-thylakoid proton gradient (ΔpH) and consequently also the thermal dissipation of excess energy (NPQ). The absence of the PSBS protein likewise prevents the formation of ΔpH-dependent NPQ. This component of NPQ is called qE, which is nearly exclusively responsible for induction of NPQ upon increase in light intensity. On the other hand, the *pgr5* mutant is not only deficient in induction of strong NPQ but it also lacks the capability to oxidize P700 upon increase in light intensity. This, in turn, results from uncontrolled electron flow toward photosystem I (PSI), which has been proposed to be caused by the lack of PSII down-regulation by NPQ and by a poor control of electron flow via the Cytochrome *b_6_f* (Cyt *b_6_f*) complex. Here we asked whether NPQ really is a component of such regulation of electron flow from PSII to PSI at high light. To this end, the two NPQ mutants *pgr*5 and *npq4*, the latter lacking the PSBS protein, were characterized. It is shown that the *npq4* mutant, despite its highly reduced Plastoquinone pool, does not inhibit but rather enhances the oxidation of P700 in high light as compared to wild type. This clearly demonstrates that the control of electron flow from PSII to PSI cannot be assigned, even partially, to the down-regulation of PSII by NPQ but apparently takes place solely in Cyt *b_6_f*. Moreover, it is shown that the *pgr5* mutant can induce NPQ in very high light, but still remains deficient in P700 oxidation. These results challenge the suggestion that NPQ, induced by PGR5-dependent cyclic electron transfer, would have a key role in regulation of electron transfer from PSII to PSI. Instead, the results presented here are in line with our recent suggestion that both PSII and PSI function under the same light harvesting machinery regulated by ΔpH and the PSBS protein (Tikkanen and Aro, 2014; Grieco et al., 2015).

## Introduction

Solar energy is converted into chemical form by photosynthetic light reactions, which in plants and green algae take place in the thylakoid membrane inside the chloroplasts. Safe and efficient function of the photosynthetic light reactions is based on synchronized function of the light-driven enzymes photosystem II (PSII) and photosystem I (PSI), the former splitting water to protons and electrons and the latter using electrons to reduce NADP to NADPH. Electron transfer from PSII to PSI takes place via the intersystem electron transfer chain composed of Plastoquinone (PQ), Cytochrome *b_6_f* (Cyt *b_6_f*), and Plastocyanin (PC). PQ accepts electrons from PSII and the electrons are then transferred to PSI via Cyt *b_6_f* and PC. The electron transfer reactions in Cyt *b_6_f* are coupled to transfer of protons from chloroplast stroma to thylakoid lumen (Q cycle). This reaction not only facilitates the generation of *trans*-thylakoid proton gradient (ΔpH) but also allows the ΔpH to control the rate of electron transfer to PSI (Joliot and Johnson, 2011; Tikhonov, 2014; Tikkanen and Aro, 2014). This is because the oxidation of plastoquinol at the Qo site is the rate limiting step of the electron transfer (Stiehl and Witt, 1969), making the rate of electron transfer dependent on ΔpH.

Photosystem II and Photosystem I have their own minor light harvesting antennae, but the energy capture to both photosystems is largely based on the major light harvesting system that is embedded in the thylakoid membrane and composed of LHCII trimers (Wientjes et al., 2013; Grieco et al., 2015). The distribution of excitation energy from the LHCII system to PSII and PSI is redox regulated (See for review: Allen et al., 1981; Murata, 2009). This regulation is based on phosphorylation of thylakoid proteins and required to maintain the functional balance between PSII and PSI upon changes in light quality (Allen et al., 1981; Mekala et al., 2015) and quantity (Mekala et al., 2015). Efficiency of the LHCII system, in turn, is regulated by ΔpH and is dependent on the PSBS protein (Li et al., 2000; Niyogi and Truong, 2013). The ΔpH generated by PSII and the Q cycle is released by ATP synthase in a reaction utilizing the proton motive force. Thus, ΔpH is eventually determined by the ratio between the ΔpH generation and release mechanisms according to the energetic state of the chloroplast (Kanazawa and Kramer, 2002; Kohzuma et al., 2013).

It is not fully understood how the regulation of *trans*-thylakoid ΔpH actually occurs according to the light intensity and the energetic state of the chloroplast. Nevertheless, it has been clearly demonstrated that strengthening of ΔpH upon increase in light intensity is dependent on proteins Proton Gradient Regulation 5 (PGR5) and Proton Gradient Regulation Like 1 (PGRL1; Munekage et al., 2002; DalCorso et al., 2008). Traditionally, the PGR5 protein is linked to the cyclic electron flow around PSI (CET) via putative Ferredoxin (FD) -PQ oxidoreductase (FQR; Munekage et al., 2002). By this mechanism, PGR5 is supposed to enhance the generation of ΔpH and thereby accelerate the induction of NPQ and slow down the Q cycle. This model, however, is paradoxical since it states that the slowdown of electron transfer (occurring in the Cyt *b_6_f* complex) results from acceleration of electron transfer via Cyt *b_6_f* complex (in CET). Based on this paradox, it has also been proposed that PGR5 simply prevents the leaking of protons from the lumen to chloroplast stroma by a still uncharacterized mechanism (Avenson et al., 2005). Whatever the mechanism, it is clear that the *pgr5* mutant cannot increase the *trans*-thylakoid ΔpH and consequently fails in the PSBS protein-dependent thermal dissipation of excess excitation energy (NPQ) upon increase in light intensity (Munekage et al., 2002; Suorsa et al., 2012). Due to the impaired control of electron transfer via Cyt *b_6_f* and low thermal dissipation of excess excitation energy, PSI of the *pgr5* mutant is sensitive for photoinhibition and has reduced amount of PSI (Munekage et al., 2002). The amount of PSI is adjusted according to the acceptor side limitation of PSI, which in turn is dependent on the light intensity, the capacity of carbon metabolism and the amount of active PSII (Munekage et al., 2002, 2008; Suorsa et al., 2012; Tikkanen et al., 2014).

Generally, NPQ is considered as a mechanism that specifically down-regulates the activity of PSII and therefore is supposed to limit electron transfer to PSI. However, based on the behavior of mutants disturbed in the distribution of excitation energy from the LHCII system to PSII and PSI (Tikkanen et al., 2010, 2011; Grieco et al., 2012), it seems highly likely that NPQ downregulates both photosystems to similar extent. Indeed, only in a specific case of the *stn7* mutant when energy distribution from the LHCII system to PSI is impaired, the relaxation of NPQ in low light selectively affects only PSII leading to high reduction of the PQ pool (Tikkanen et al., 2010, 2011; Grieco et al., 2012). This challenges the idea that NPQ is required for oxidation of P700 in high light. Moreover, it has been reported that PSBS mutants can oxidize P700 upon increase in light intensity (Grieco et al., 2012; Roach and Krieger-Liszkay, 2012) indicating that the PSBS-dependent mechanism does not specifically downregulate PSII, but rather affects both photosystems. Previously the interaction between proton gradient-dependent regulation of electron transfer and NPQ was studied in *Chlamydomonas reinhardtii* (Kukuczka et al., 2014). It was shown that the two mechanisms are complementary, both of them being needed for high light acclimation in oxygen limiting conditions.

Here, we demonstrate that the PSBS protein- and ΔpH-dependent NPQ are needed to prevent over-reduction of the PQ pool at high light, but importantly, NPQ is not required for oxidation of P700 at high light. On the contrary, the oxidation of P700 at high light is even enhanced in the *npq4* mutant as compared to wild type (WT), indicating that in the absence of PSBS-dependent NPQ the excitation energy transfer to PSI is enhanced. This also points out that the deficiency of the *pgr5* mutant to oxidize P700 cannot result from the deficient NPQ, but more likely solely from the missing photosynthetic control via Cyt *b_6_f*.

## Materials and Methods

Wild type (ecotype Columbia) and mutant lines *pgr5* (Munekage et al., 2002) and *npq4* (Li et al., 2000) of *Arabidopsis thaliana* were grown at 23°C and in 60% relative humidity under an 8-h photoperiod of constant white light (110–120 μmol photons m^-2^ s^-1^) with OSRAM PowerStar HQIT 400/D Metal Halide lamps as a light source. Leaves from 5-weeks-old plants were used for the experiments. Detached leaves with petioles submerged in tap water were incubated 10 min in darkness before the measurements. For each lineage, leaves from three different plants were analyzed and SD was calculated with formula $\sqrt{\sum {\left(x-\bar{x}\right)}^{2}/(n-1)}$.

Chlorophyll *a* fluorescence and signal from oxidized P700 (Klughammer and Schreiber, 1994, 2008) were detected with Dual-PAM-100 (Heinz Walz). A saturating pulse (5000 μmol photons m^-2^ s^-1^ for 500 ms) was applied in every 1 min with increasing 635-nm actinic light (50, 127, 274, 661, and 1595 μmol photons m^-2^ s^-1^ or 127 and 1953 μmol photons m^-2^ s^-1^). Chlorophyll *a* fluorescence was detected with 460-nm measuring light (19 μmol photons m^-2^ s^-1^) and oxidation state of P700 was determined based on the difference of intensities 875 nm and 830 nm of pulse-modulated measuring light reaching the photodetector (Klughammer and Schreiber, 2008). PSI redox state (*P*/*P*_m_) was obtained by normalizing the signal of oxidized P700 at a given light phase (P) to the signal of maximal proportion of oxidized P700 under a saturating pulse with far-red background (*P*_m_; Klughammer and Schreiber, 1994). Relative reduction of *Q*_A_ (*F*′/*F*_m_) was determined by normalizing the fluorescence under actinic light (*F*′) to the maximal fluorescence of dark-adapted leaf (*F*_m_). Induction of NPQ (1–*F*_m_′/*F*_m_) was calculated by reversing the maximal fluorescence from a light-adapted leaf (*F*m′) normalized to the maximal fluorescence of a dark-adapted leaf (*F*_m_).

## Results

**FIGURE 1:**
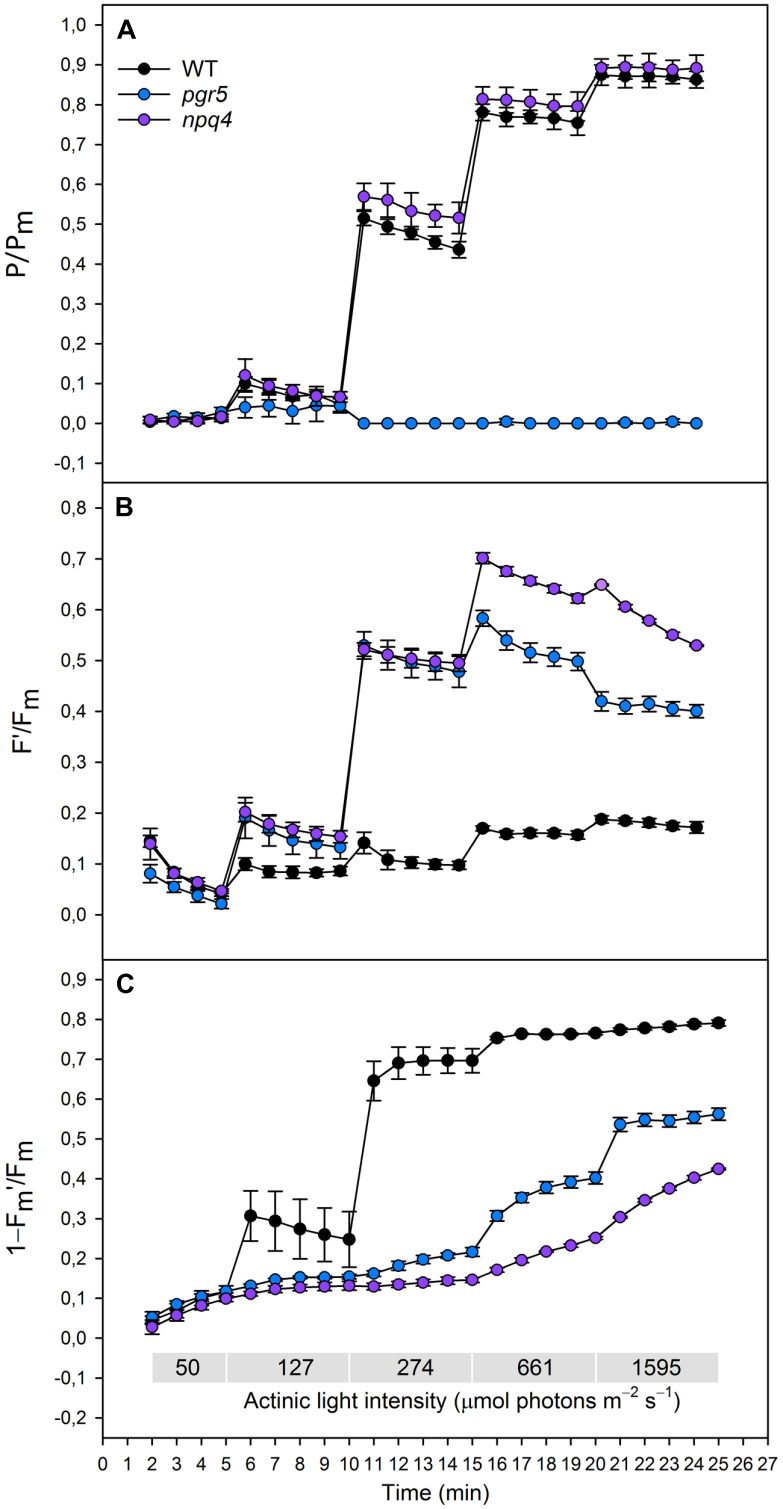
**(A)**(P/P_m_), **(B)** (*F*′/*F*_m_), **(C)** (1–*F*_m_′/*F*_m_) in wild type (black dots), *pgr5* (blue dots), and *npq4* (purple dots) during a stepwise increase in actinic light intensity. Saturating pulse was applied in every 1 min with gradually increasing actinic light intensity (50, 127, 274, 661, and 1595 μmol photons m^-2^ s^-1^). Detached leaves were incubated in darkness 10 min before measurements. Representative data is shown with three different plants measured of each lineage.

**FIGURE 2:**
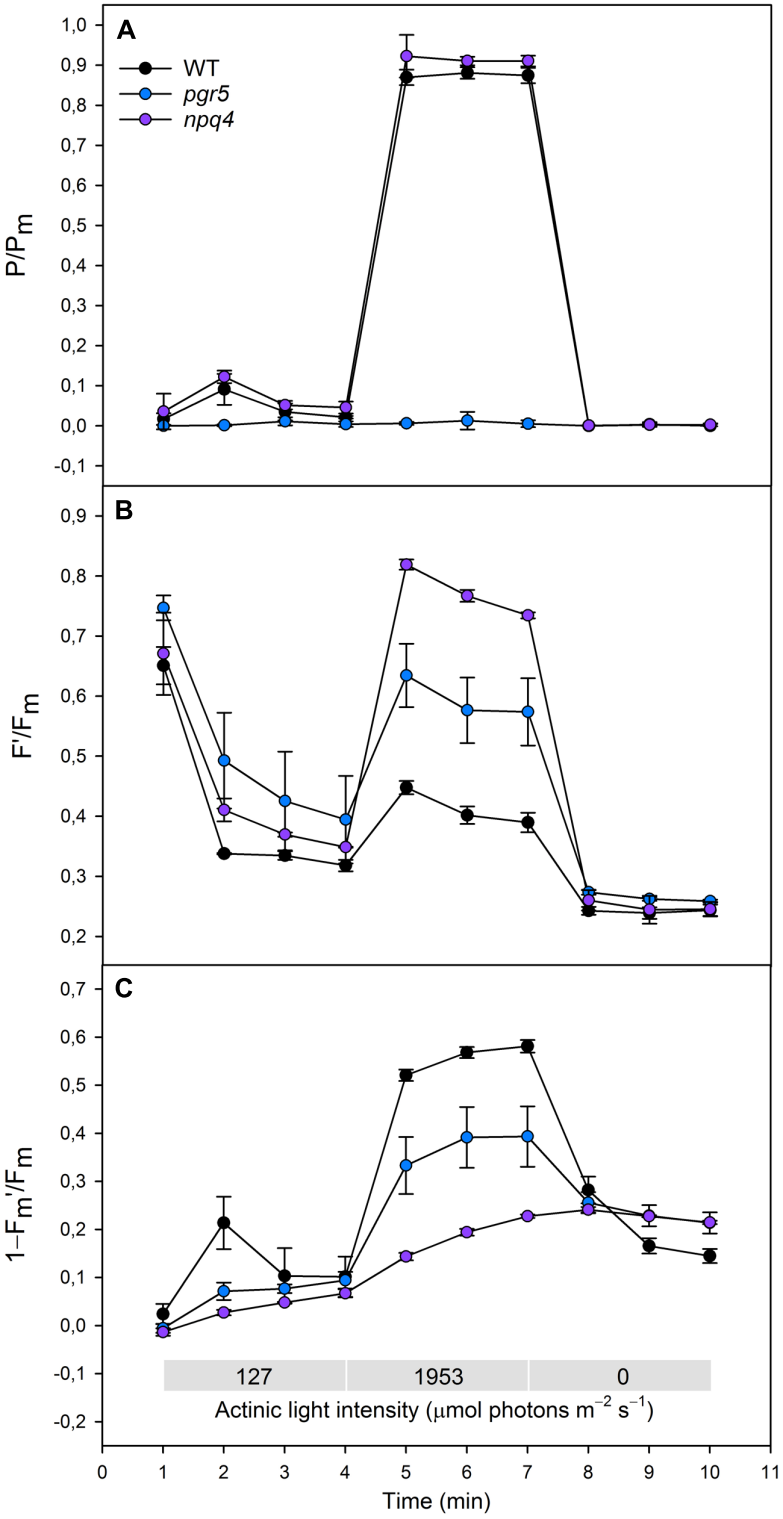
**(A)**(P/P_m_), **(B)** (*F*′/*F*_m_), **(C)** (1–*F*_m_′/*F*_m_) in WT (black dots), *pgr5* (blue dots), and *npq4* (purple dots) during a steep increase in actinic light intensity. Saturating pulse was applied in every 1 min with a non-gradual increase in the actinic light intensity (0, 127, and 1953 μmol photons m^-2^ s^-1^). Detached leaves were incubated in darkness 10 min before measurements. Representative data is shown with three different plants measured of each lineage.

To clarify the differential roles of ΔpH-dependent control of Cyt *b_6_f* and NPQ in regulation of electron flow from PSII to PSI at high light, we investigated WT, *pgr5*, and *npq4* with respect to the redox state of PSI, redox state of PSII electron acceptors and induction of NPQ upon changes in illumination conditions. The functional phenotypes of the mutants were addressed by applying saturating pulses with actinic light intensity increasing either gradually (50, 127, 274, 661, and 1595 μmol photons m^-2^ s^-1^; **Figure 1**) or in two steps from darkness to light slightly higher that growth light (127 μmol photons m^-2^ s^-1^) and subsequently to very high light (1953 μmol photons m^-2^ s^-1^; **Figure 2**).

Redox state of PSI was determined by normalizing the signal of oxidized P700 to the signal of maximal proportion of oxidized P700 (*P*/*P*_m_). When increasing the actinic light intensity stepwise, the *npq4* mutant showed a higher oxidation level of P700 at moderate high light intensities (274 and 661 μmol photons m^-2^ s^-1^), whereas no difference between *npq4* and WT was detected at lower or higher intensities (**Figure 1A**). The steep increase in actinic light intensity, on the other hand, resulted in more substantial difference in the oxidation of P700 between *npq4* and WT (**Figure 2A**). In *pgr5*, P700 oxidized slightly during the low actinic light intensities (50 and 127 μmol photons m^-2^ s^-1^), but remained reduced under higher intensities (**Figure 1A**) and throughout the drastic increase of actinic light intensity (**Figure 2A**).

To study the redox state PSII acceptor side, the redox state of Q_A_ was estimated by normalizing fluorescence to the maximal fluorescence (*F*′/*F*_m_). Although the fluorescence normalized to the maximal fluorescence does not linearly correlate with [*Q*_A_^-^] due to the antenna connectivity (Lavergne and Trissl, 1995; Joliot and Joliot, 2003), the parameter *F*′/*F*_m_ is the best parameter for the mutants with severely altered behaviour of both *F*_m_′ and *F*′. During the gradual increase of actinic light intensity, WT maintained its *F*′/*F*_m_ level, whereas *pgr5* and *npq4* showed a drastic increase in the parameter already at light slightly higher that growth light and throughout the experiment (**Figure 1B**). In addition, *npq4* reached a still higher level of *F*′/*F*_m_ than *pgr5* at the high light intensities (661, 1595 μmol photons m^-2^ s^-1^), during which the *Q*_A_ begun to return to its oxidized state (**Figure 1B**). The differential reduction pattern of *pgr5* and *npq4* recurred during the steep increase of actinic light intensity (**Figure 2B**).

Induction of NPQ (1–*F*_m_′/*F*_m_) was analyzed in order to clarify the relationship between thermal dissipation of excess excitation energy and redox state of ETC. NPQ was almost non-existent in the *npq4* mutant, whereas *pgr5* was capable of inducing a relatively high NPQ compared to *npq4* especially in very high light (**Figures 1C and 2C**). In addition to the slower induction, both mutants showed an impaired relaxation of NPQ during the subsequent phase of darkness (**Figure 2C**).

## Discussion

**FIGURE 3:**
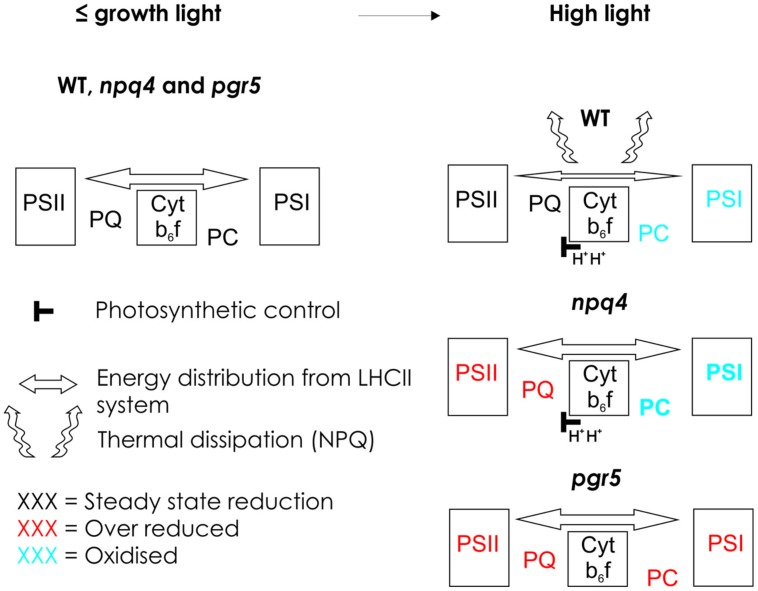
**Schematic model presenting the redox state of electron transfer chain in WT and in *npq4* and *pgr5* mutants in growth light and in high light.** In growth light or light intensities below the growth light, WT, *npq4*, and *pgr5* can keep the intersystem electron transfer chain optimally oxidized. Increase in light intensity enhances ΔpH in WT and *npq4* but not in *pgr5*. Increased ΔpH slows down electron flow via Cyt *b_6_f* in WT and *npq4*, but induces NPQ only in WT. In WT, NPQ prevents the over-reduction of Plastoquinone (PQ) pool and slows down the Cyt *b_6_f* leading to oxidation of plastocyanin (PC) and photosystem I (PSI). In the *npq4* mutant, with low NPQ but normal photosynthetic control, high light leads to high reduction of PQ pool, and enhanced oxidation of PC and PSI. This indicates that in the absence of PSBS protein-dependent NPQ, the photochemical capacity of the both photosystems in improved. In the *pgr5* mutant, that cannot raise the ΔpH upon increase in light intensity, the entire electron transfer chain becomes over-reduced. This results from the incapability to slow down the Cyt *b_6_f* rather than from the low level of NPQ.

Limitation of electron flow to PSI upon increase in light intensity has been shown to be crucial for protection of PSI against photodamage (Munekage et al., 2002; Suorsa et al., 2012). The mechanisms involved in such a regulation of electron flow have, however, remained elusive. Here, we compared the putative effects of NPQ and the reduction state of the PQ pool on P700 oxidation in WT and in the *pgr5* and *npq4* mutants (Schematic model, **Figure 3**). It has been proposed that the deficiency of *pgr5* in generation of ΔpH upon increase in light intensity is due to incomplete cycling of electrons from PSI acceptor side back to the PQ pool by a putative FQR (Munekage et al., 2002). This obviously has not been considered to be a problem because the simultaneously induced energy-dependent NPQ is believed to selectively downregulate PSII, thus leading to PSII limitation of electron transfer, and consequent oxidation of PSI. We tested this hypothesis and demonstrated (**Figure 1**) that despite the absence of NPQ, the *npq4* mutant perfectly oxidizes P700, even more efficiently than WT. Importantly, P700 is oxidized (**Figures 1A and 2A**) despite the fact that the PQ pool is at the same time strongly reduced (**Figures 1B and 2B**). The concomitant reduction of the PQ pool and oxidation of P700 strongly suggest that the electron transfer is controlled by Cyt *b_6_f*. Moreover, the fact that oxidation of P700 is facilitated in the *npq4* mutant indicates that in the absence of the PSBS protein, PSI has more excitation energy to oxidize P700 as compared to WT. This difference, however, decreases at extremely high light (**Figure 1A**), indicating that when the light intensity is strong enough, the capacity of PSBS-dependent quenching mechanism to limit excitation pressure becomes saturated.

The *pgr5* mutant is severely deficient in induction of NPQ when the increase in light intensity is not extreme (**Figure 1C**). Nevertheless, when the increase in light intensity is strong enough, the *pgr5* mutant can induce a reasonably high NPQ that in WT occurs concomitantly with oxidation of P700 (**Figure 2A**). Despite the induction of NPQ, the *pgr5* mutant cannot oxidize P700 (**Figures 1A and 2A**), which further confirms that NPQ is not a mechanism to control the electron flow to PSI. Further support to this conclusion is provided by experiments (Tikkanen et al., 2010; Grieco et al., 2012) conducted with the *stn7* mutant deficient in excitation energy transfer to PSI. Comparison of WT and *stn7* with respect to the reduction state of the electron transfer chain, as affected by both the induction and relaxation of NPQ, revealed two distinct phenomena. First, the redox state of the PQ pool in WT remains relatively stable despite the induction or relaxation of NPQ. Second, in the *stn7* plants, the relaxation of NPQ leads to reduction of the PQ pool (Tikkanen et al., 2010; Grieco et al., 2012). Taken together, it can be concluded that when the excitation energy distribution from the LHCII system to PSII and PSI is in balance, NPQ does not change the relative capacity of PSII and PSI electron transfer (Tikkanen et al., 2011). It is worth noting here that opposite to the independence between NPQ and oxidation of PSI, already a moderate photoinhibition of PSII leads to selective down-regulation of PSII and consequent oxidation of PSI (P700; Tikkanen et al., 2014).

Importantly, *pgr5* is more efficient in oxidation of P700 in low light than in high light (**Figure 1**). This may suggest that in the absence of PGR5-provided ΔpH and resistance against proton extrusion from the lumen, the NDH-1-dependent cyclic is enhanced. Similar to bacterial and mitochondrial complex I (Efremov et al., 2010), a transfer of electron is coupled with translocation of four protons into thylakoid lumen via the NDH-1 complex (For a review: Battchikova et al., 2011; Kramer and Evans, 2011). This increases the amount of translocated protons in relation to transported electrons as compared to linear electron transfer and the FQR-CET. The additional ΔpH generated by NDH-1-CET may increase the resistance against LET via Cyt b_6_f, leading to enhanced oxidation of P700 in *pgr5* in low light. Oxidation of P700 is, however, lost when the actinic light exceeds the intensity of growth light. This indicates that the NDH-1-dependent protonation of lumen is not capable of compensating the function of the PGR5 protein in high light. Indeed, in high light the PGR5 protein is essential in controlling the rate of the intersystem electron transfer.

In our opinion, based on the facts that NPQ plays no role in oxidation of P700 *in vivo* and the FQR-CET model is paradoxical in requiring simultaneous acceleration and deceleration of the electron transfer via Cyt b6f, it seems highly unlikely that the function of the PGR5 protein in PSI CET is to keep P700 oxidized. Therefore, we assume that there is a still uncharacterized PGR5-dependent mechanism that controls proton translocation across the thylakoid membrane and allows synchronized induction of NPQ together with Cyt *b_6_f*-dependent mechanism to control electron flow to PSI. A good candidate for such a mechanism is the regulation ATP synthase according to the redox state of electron transfer components between light reactions and carbon assimilation reactions (Kohzuma et al., 2013). It is known that PGR5 increases the resistance against proton translocation from thylakoid lumen to chloroplast stroma (Avenson et al., 2005). Interestingly, the PGR5-PGRL1 complex has redox active thiol groups being able to accept electrons from ferredoxin (Hertle et al., 2013). It is conceivable that the PGR5-PGRL1 complex senses the redox state of PSI electron acceptors and accordingly exerts feedback-regulation on photosynthetic light reactions, by tuning the resistance of proton translocation via ATP synthase by a mechanism that remains to be characterized.

## Conflict of Interest Statement

The authors declare that the research was conducted in the absence of any commercial or financial relationships that could be construed as a potential conflict of interest.
